# Priority setting for health technology adoption at the national level: Lessons learned over 25 years’ experience

**DOI:** 10.1017/S0266462323002611

**Published:** 2023-11-06

**Authors:** Osnat Luxenburg, Tal Morginstin, Vicki Myers, Mor Saban, Joshua Shemer, Rachel Wilf-Miron

**Affiliations:** 1 Medical Technology, Health Information and Research Directorate, Ministry of Health, Jerusalem, Israel; 2 Gertner Institute of Epidemiology and Health Policy Research, Sheba Medical Center, Ramat Gan, Israel; 3 Assuta Medical Centers, Tel Aviv, Israel; 4 School of Public Health, Faculty of Medicine, Tel Aviv University, Tel Aviv, Israel; 5 Department of Nursing, School of Health Professions, Faculty of Medicine, Tel Aviv University, Tel Aviv, Israel

**Keywords:** technology assessment, prioritization, health service basket, Israel

## Abstract

**Background:**

Limited health budgets and continual advancement of health technologies require mechanisms for prioritization. Israel, with a publicly funded health service basket, has implemented and optimized such a health technology assessment process since 1999.

We describe the process of evaluating technologies according to the Israeli model, analyze its outputs and benefits over two decades of implementation, and compare its key features with international experience.

**Methods:**

Retrospective data were collected between 1998 and 2023, including work processes, committee composition, number of applications submitted and approved by a clinical domain, and yearly cost of the basket. Features were evaluated within the evidence-informed deliberative process (EDP) framework.

**Results:**

This national model involves relevant stake holders in a participatory and transparent process, in a timely manner, and is accepted by the public, health professionals, and policy makers, facilitating early adoption of the newest medical technologies. Between 11 and 19 percent of applications are approved for reimbursement annually, mostly pharmaceuticals. On average 26 percent of approved technologies are added to the list without additional budget. Major domains of approved technologies were oncology, cardiology, and neurology.

**Conclusions:**

Israel created a unique model for the expansion of the health service basket. Despite an increasing number of applications and rising costs, the mechanism enables a consensus to be reached on which technologies to fund, while remaining within budget constraints and facilitating immediate implementation. The process, which prioritizes transparency and stake holder involvement, allows just a resource allocation while maximizing the adoption of novel technologies, contributing to an outstanding national level of health despite relatively low health spending.

## Introduction

The adoption of new health technologies within budgetary constraints is one of the major challenges for healthcare systems globally. The rapid evolution in the field of medicine exerts pressure on healthcare systems to add new technologies to their existing basket of services in the diagnostic, preventive, therapeutic, and rehabilitative domains (1).

Total expenditure on healthcare has risen considerably over the last decades across all OECD countries (2). This growth has been predicted to continue in the coming years due to the aging population, the introduction of innovative, costly and effective technologies, and rising public expectations (3;4). Given these trends, in the reality of limited resources, policy makers are required to decide which new technologies to publicly fund and which to reject or postpone (5).

Health technology assessment (HTA) was defined in 2020 in the context of an international collaboration as “a multidisciplinary process that uses explicit methods to determine the value of a health technology at different points in its lifecycle. The purpose is to inform decision making in order to promote an equitable, efficient, and high-quality health system*”* (6). Health technologies include, among others, tests, devices, medicines, vaccines, procedures, programs, or systems. HTA synthesizes evidence on safety, efficacy, and economic evaluation and presents it to decision makers – both clinicians and administrators – in order to promote an equitable, efficient, and high-quality health system.

Historically, healthcare services in Israel have been developed by four not-for-profit health maintenance organizations (HMOs), originally called “sick funds” (7). The Israeli National Health Insurance (NHI) Law, enacted in 1995, determines a basic list of diagnostic, preventive, curative, and rehabilitative health services to be publicly funded and provided by the four HMOs which citizens can choose from to provide their healthcare (8). Since the enactment of the law, the entire Israeli population has been insured via one of these four HMOs (9). The government distributes the NHI budget among the HMOs primarily through a weighted capitation scheme. The organizational, information technological, and logistical capacities of Israel’s community-based healthcare providers, that is, the HMOs, were recently mentioned as major contributors to the rapid rollout of vaccination for COVID-19 (10).

Since the law does not specify a “technological coefficient” for automatic financial updates in accordance with technological advances, the addition of new technologies to the national list of publicly funded health services requires a yearly budgetary allocation, anchored in the Annual State Budget. The inevitable tension between the demand for advanced technologies and the limited availability of public resources called for mechanisms for prioritization of medical technologies that give the greatest benefits to society, in order to maximize population health within the predefined budget (5;11). Since 1998, a continuous multidisciplinary process has been led by the Medical Technology, Health Information and Research (MTIR) Directorate at the Israeli Ministry of Health (MOH) which is one of the longest-running national HTA systems (12;13).

Several frameworks have been suggested for evaluating HTA systems. Angelis et al. (14) included four elements (i) responsibilities and structures of HTA agencies, (ii) evidence and evaluation criteria, (iii) methods and techniques applied, and (iv) outcomes and implementation.

Oortwijn et al. (15) proposed a framework using evidence-informed deliberative processes (EDPs) for HTA which comprises eight steps of EDPs (i) installing an advisory committee, (ii) defining decision criteria, (iii) selecting health technologies for HTA, (iv) scoping, (v) assessment, (vi) appraisal, (vii) communication and appeal, and (viii) monitoring and evaluation and four critical elements (i) stake holder involvement, (ii) evidence-informed evaluation, (iii) transparency (the deliberative processes, including objectives, modes of stake holder involvement, and the decision reached and its related argumentation, explicitly described and made publicly available), and (iv) the element of appeal, which ensures that a decision can be challenged and revised if new information or insights become available.

We aim here to describe the process of evaluating technologies according to the Israeli model, relating to the EDP framework, analyze its outputs and benefits and summarize the lessons learned for more than two decades since implementation, and compare its key features with international experience.

## Methods

### Study design and variables

Description of the Israeli national model for updating the list of publicly funded health services was based on the data obtained from the MTIR directorate including a detailed analysis of process documentation.

A retrospective archive study was conducted. Data were collected between the years 1998 and 2023 and included the following parameters: information on the stages of the process including the meetings and decisions of the National Public Committees, number of proposals submitted and applications approved each year, the budget allocated for new technologies each year as well as the yearly (cumulative) budget of the national list of reimbursed health services, and the proportion of the budget by the clinical domain of approved applications.

A comparison was conducted between key features of the Israeli system with other countries based on the EDP framework.

## Results

### The Israeli model for priority setting of health technology adoption

The Israeli mechanism for updating the national list of publicly funded health services is led by two main bodies (i) a permanent group of professional experts - the Health Technologies Forum (HTF) at MTIR - and (ii) an ad hoc Public National Advisory Committee. The committee members (in the last decade between 17 and 20 members) are appointed for one year by the Ministers of Health and Finance, with representatives from diverse sectors including all four HMOs, leading medical professionals (four members), four economists – usually two from the Ministry of Finance and two from the Ministry of Health – and independent public representatives from the fields of religion, ethics, social sciences, and welfare (4), as well as a chairperson and secretary of the committee.

**Figure 1.**
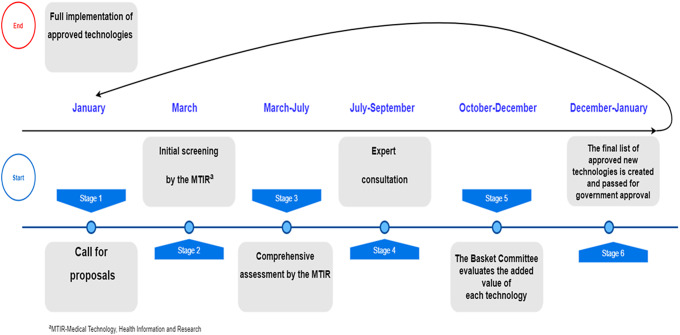
The annual cycle of updating the national list of reimbursed health services – The Israeli model.

The two-stage process is illustrated in Figure 1. Stage 1: **Call for proposals and processing of applications by HTF**: An open call for applications relating to the needs of the healthcare system is published by the Director General of the Ministry of Health, open to all, including professional bodies, patient interest groups, individual citizens, pharmaceutical and medical device companies, hospital staff, and politicians. Submissions can include medications, medical devices, diagnostic and imaging techniques, health services, screening tests, and surgical and other invasive procedures. All applications are screened for added clinical value, reviewing the latest available evidence. Furthermore, each application goes through a thorough workup by the professional team to bring it to a uniform standard so that when it comes to the committee it cannot be identified if it came from a private entity or a professional organization. This assures anonymity of the submitting source for decision makers and all parties involved in the process, except for the professional team members who are in dialogue with the submitters when clarification is required. The MTIR professionals conduct a comprehensive assessment of all submitted applications, in a predetermined format. This assessment is based on clinical, legal, economic, ethical, epidemiological, and policy considerations and results in a list of technologies to be presented to the public committee. This is an example of evidence-informed evaluation, the second element of the EDP framework. HTF further consults with medical experts regarding technologies that pass the initial screening and undergo a more comprehensive review. This consultation adds input from the clinical field, beyond the facts and figures available from the scientific literature. Experts are required to rate the applications according to three professional scales (a) clinical benefit compared with the evidence-based current standard, (b) acceptability of the technology’s use, in Israel and abroad, and (c) the importance of including the technology in the list of services. Once rated, a table is created, ranking the technologies in order of importance. Public appeals relating to certain technologies are included in the materials submitted to the public committee. These include personal testimonies of patients who have used the technology, whether through private insurance or in clinical trials, to provide patient experience, beyond clinical details.

Stage 2: **Assessment by the Public National Advisory Committee**. The Public Committee evaluates the added value of each of the hundreds of technologies submitted each year, to be considered for addition to the publicly funded list of services. Applications undergo preliminary grading by importance - high, medium, or low priority. This classification guarantees that the chance of a technology being approved is not influenced by the order in which it is presented. Each technology goes through several rounds of deliberation. The technologies rated highest continue to the next round so that the final list contains the technologies ranked with the highest importance. In the early stages, technologies are rated on clinical criteria, to what extent they provide added value compared to existing options; at the later stages, cost enters the evaluation. The selection of new technologies is based on guiding criteria related to clinical, epidemiological, and cost issues while considering any relevant ethical, social, and legal issues. The criteria used by the Public Committee are mostly universal, have been previously published (5;11), and include issues such as need, appropriateness, and clinical benefits, epidemiological criteria such as incidence and prevalence of the disease and the estimated target population, assessment of scientific evidence for clinical efficiency, comparison to alternative existing treatments, life extension and quality of life both regarding improved function and reduced pain/suffering; evaluation of the predicted annual cost of treatment taking into account the savings from not needing the existing therapy; equality, solidarity, and other ethical or social values and more. The Public Committee is mandated to resolve conflicts and trade-offs between competing moral principles based on the value judgments of its members. For example, sometimes acute needs for high-cost treatment in a small group conflict with chronic needs on a wider population scale. Decisions are made by consensus among all committee members.

The final list of all approved technologies is manifested as a clear, detailed, unambiguous list, ready for nationwide implementation.

The whole process is mostly transparent, with applications, decisions, budget allocation, and committee protocols all made public (since 2008, with the exception of the COVID-19 pandemic years when some protocols were not published due to logistic issues). Full transcripts of the meetings are published on the MOH website. The committee’s decisions are published on the MOH website, shortly after each meeting, to allow anyone to appeal. Decisions are made not only considering the cost but also taking into account the ethical and social factors, for example, a high-cost treatment for a rare condition may be approved even though the same budget could cover treatment for many more patients with a more prevalent disease. Furthermore, over the years, there has been an ongoing effort to balance between acute medical needs (such as oncology drugs) and chronic population-wide issues (e.g., preventive medicine and diabetes care), taking into consideration clinical, economic, ethical, and social factors, and data on the size of the affected population, the costs to be incurred, and the effect of not funding the treatment. The entire healthcare system is involved in the process, increasing the acceptability of the resulting decisions and their implementation, usually within two weeks after the committee’s decision is approved by the Israel Health Council and the government.

Over the years, the process has evolved and adapted to bridge the gap between the fast-growing costs of new technologies and the limited budget allocated for the adoption of new technologies. An example of this adaptation is the risk-sharing mechanism, by which some of the financial risks of adopting new technologies are shifted from the payer (HMO) to the technology sponsor (i.e., pharmaceutical company). This balances the technology sponsor’s desire for early market access with the necessity to ensure effective and efficient use of limited healthcare resources. Risk sharing has been shown to improve the efficiency of utilization of healthcare services. An analysis of risk-sharing agreements between 2011 and 2018 found that in 44 percent of said agreements, actual utilization exceeded the pre-specified threshold leading to the HMOs receiving reimbursement from pharmaceutical companies (16). Marketing authorization holders may submit risk-sharing models, such as managed entry agreements (17), which are brought for consideration before a subcommittee and the best model chosen. Computerized data from the HMOs on the degree to which the new technologies are implemented and utilized, and the budget allocated for technologies used, allows for periodic monitoring and fine-tuning. This enables optimization of the allocated budget for the future inclusion of additional technologies or for the substitution of underutilized new technologies with alternative treatments for the same group of patients.

**Figure 2.**
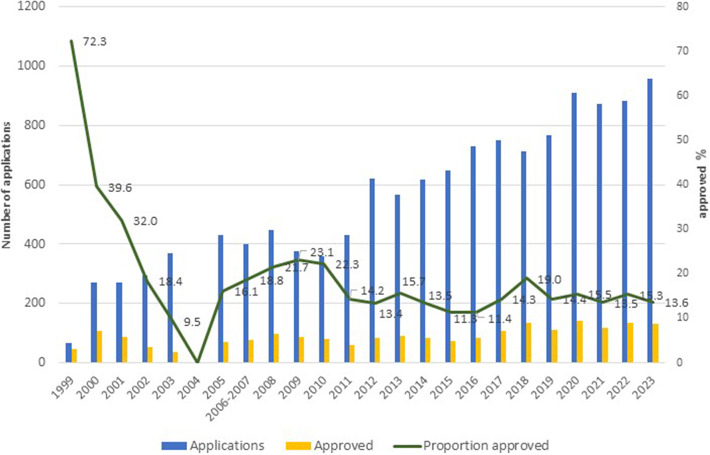
Number of applications submitted and approved, 1999–2023* ** *Between the years 1999 and 2002, data were only available for pharmaceutical applications and approvals, which comprised 80–85 percent of applications. **Missing data in 2004, the single year in the study period when no budget was allocated for the enlargement of the health service basket.

Figure 2 presents the number of technologies submitted at the call for proposals and the number of technologies approved each year, as well as the proportion. The number of requests has increased steadily over the years. Approximately 70 percent of initial submissions are deliberated by the committee having passed preliminary screening. Initially, with a small number of applications, a higher proportion was approved, but with the rise in submitted applications, this proportion stabilized in the last decade around 11–19 percent. Around 50 percent of pharmaceutical applications are new submissions, around a third are re-submissions, and the rest are applications to expand the existing inclusion criteria of the list.

The budget allocated for reimbursed new health technologies has gradually increased over the years, from 150 million New Israeli Shekels (NIS) (around $42 million) in 1998, the first year of prioritization (exchange rate 1USD = 3.6 shekels) to 650 million NIS (around $186 million) in 2023 (exchange rate 1 USD = 3.5 shekels), with the cost of new technologies being added to the base budget for health services.

Since 1998, a yearly additional budget has been allocated by the Government for updating the national list of reimbursed health services, while the committee recommends which new technologies will be covered by this amount. The allocation for expanding the list varies between 0.8 and 1.7 percent of the entire health service basket and has stabilized in recent years around 0.9 percent.

Over the last two decades, medications comprised the majority of new technologies added to the list. Non-pharmaceutical technologies such as imaging or medical devices comprised 13–40 percent, on average around one-fifth of approved submissions.

**Figure 3.**
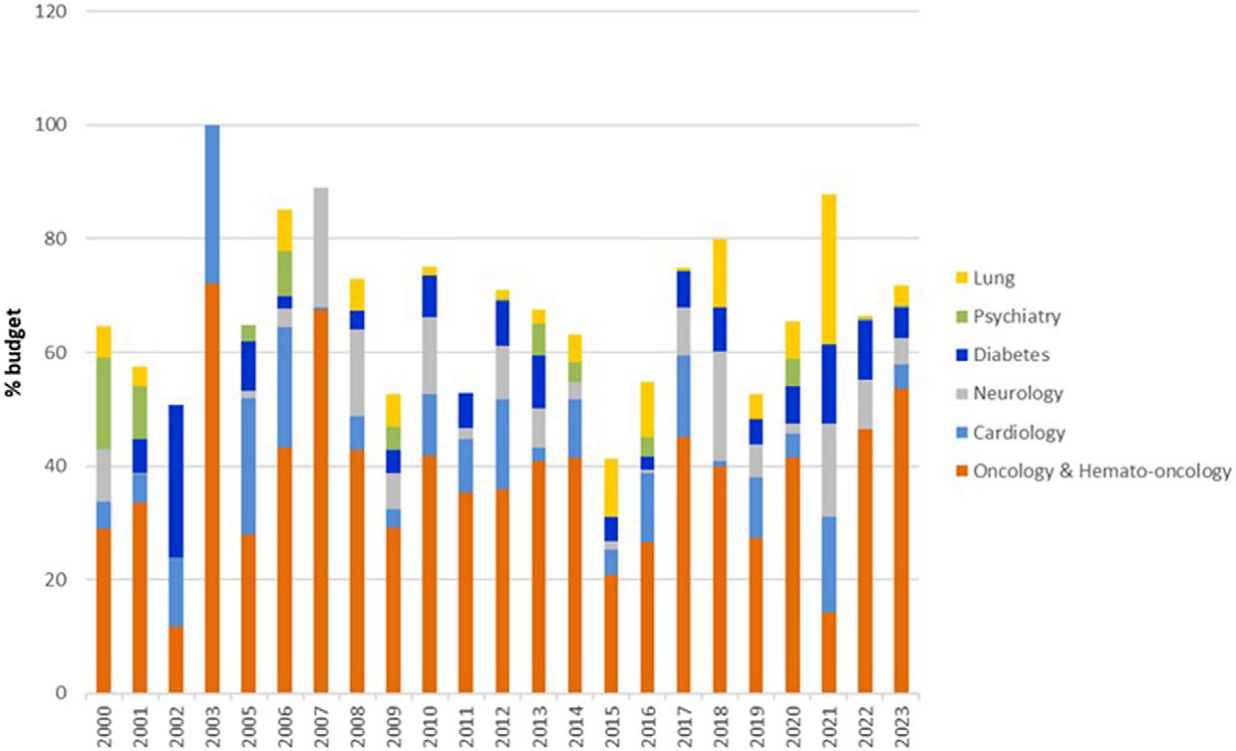
Proportion (%) of the budget by clinical domain for approved technologies*, 2000–2023. *includes the six largest domains.

Figure 3 presents the proportion of the budget by clinical domain of approved applications. Oncology (including hemato-oncology) comprised the largest number and proportion of approved technologies throughout 1998–2023 (mean 38 percent), followed by cardiovascular (10 percent), neurology (7 percent), and endocrinology (mainly diabetes 6 percent). The cost of oncology drugs approved by the committee has risen exponentially, from an average yearly cost of approximately 60,000 NIS ($18,000) per patient per new drug in 2009 to over 130,000 NIS ($43,000) in 2018 (figures adjusted for inflation). Some fields, such as mental health, had more applications approved in the early years and far fewer in recent years. Fields that have seen an increase in requests approved in recent years include rehabilitation, dental health, imaging, and genetic testing.

Each year some technologies - on average 26 percent of the number of new technologies approved - are added to the list without an additional allocated budget. Most of these are new medications that replace older versions of the same therapeutic group and whose budget is already accounted for. Some are new medications that reduce the need for other healthcare services, thus offsetting costs that would be spent by the same HMO and avoiding the need for additional budget. In other cases, a new therapy replaces a more complicated and costly therapy, such as percutaneous transluminal coronary angioplasty that replaced cardiac surgery.

Table 1 summarizes the key characteristics of the Israeli model.

**Table 1.** Key features of the Israeli model following the EDP framework

Stake holder involvement	Involvement of a broad range of stake holders, including medical, economic, ethics experts, and citizens, is central to the process, and includes the 7Ps (18), all involved at some point of the process (i) patients and the public (who can submit applications and appeals, and patients provide personal written testimony to the committee), (ii) providers, (iii) purchasers, (iv) payers - all three (ii, iii, and iv) are represented by the HMOs in Israel and the Ministry of Health also as a payer, (v) policy makers (Ministries of Health and Finance), (vi) product makers (pharmaceutical and medical technology companies who can submit applications), and (vii) principal investigators/researchers who provide feedback from trials that have yet to be published or provide further detail on experience with the technology in Israel. This stake holder involvement is critical for success, and to ensure the engagement of all relevant groups, enabling high-quality information to be obtained to support prioritization and allowing for immediate and successful implementation of new technologies.
Evidence-informed evaluation	A comprehensive assessment is conducted of all submitted applications, in a predetermined format. This assessment is based on clinical, legal, economic, ethical, epidemiological, and policy considerations, incorporating the most up-to-date scientific data. HTF further consults with medical experts regarding technologies that pass the initial screening and undergo a more comprehensive review. This consultation adds input from the clinical field, beyond the facts and figures available from the scientific literature. Experts are required to rate the applications according to three professional scales (a) clinical benefit compared with the evidence-based current standard, (b) acceptability of the technology’s use in Israel and abroad, and (c) the importance of including the technology in the list of services. Selection of new technologies is based on guiding criteria related to clinical, epidemiological, and cost issues while considering any relevant ethical, social, and legal issues. The Public Committee considers issues such as need, appropriateness, and clinical benefits, epidemiological criteria such as incidence and prevalence of the disease and the estimated target population, assessment of scientific evidence for clinical efficiency, comparison to alternative existing treatments, life extension, and quality of life both regarding improved function and reduced pain/suffering; evaluation of the predicted annual cost of treatment taking into account the savings from not needing the existing therapy; equality, solidarity, and other ethical or social values; and more.
Transparency	Over the years, transparency evolved and increased. Applications, decisions, budget allocation, and committee protocols are all made public. Details regarding the process, participants, prioritization criteria, and the list of submitted technologies are available online. The committee’s decisions are published on the MOH website, shortly after each meeting, allowing the submission of appeals. After the discussion of appeals and publication of the decisions regarding those appeals, the committee meets for a final “marathon” of decisions over several days. Results are published as a short summary on the website within two working days. Full transcripts of the committee meetings are published on the MOH website after the final decision, with only speakers’ names and personal details redacted. All consultants and committee members are required to declare any conflicts of interest. Any potential conflicts declared by committee members are investigated and published online alongside the legal consultation regarding each disclosed conflict. Transparency contributes to the acceptance of the process by both professionals and the public.
The element of appeal	There are two junctions when appeals can be made: (1) Anyone can submit an appeal during the process (since mid-process decisions are published online) and the committee has a session dedicated to discussing the appeals. All appeals are discussed and taken into consideration before the committee issues its final recommendations. (2) After the final decision has been made and published in the MOH website, appeals can then be made via the legal system. Applications which are not approved can be resubmitted the following year. Submitters can choose to meet, at no charge, with the professional MTIR staff to prepare and revise their submissions.

## Comparison with other countries

Many countries include some similar features in their process. The Israeli model is characterized by the comprehensive inclusion of these key features and the proven experience over more than two decades. Here, we compare the six steps of the decision-making process between Israel and other countries, including but not limited to those described by Oortwijn et al.’s comparison of HTA bodies around the world (15):

- **Installing an advisory committee:** Regarding the broad spectrum of stake holders involved in the process, this varies across countries – while in Israel the committee is diverse and aims to maximize the involvement of stake holders, some countries involve only scientific experts in the process, and others additionally involve patients, members of the public, or healthcare professionals (Canada and Australia) while some bodies consult only with government representatives, for example, Greece, which has been criticized for not sufficiently involving patients, clinicians, or manufacturers in the process (19). The Canadian Drug Expert Committee, which recommends which drugs will be included in the publicly funded drug plan, is similar to the Israeli committee, consisting of 16 members with voting rights including one chair, three patient representatives, one ethicist, and 11 expert members - physicians, economists, and pharmacists. Although the mandate of the committee is advisory in Israel, as in Brazil, France, Thailand, Canada, Scotland, and Australia, in practice, the Minister of Health has always approved the committee’s recommendations. In Germany and the United Kingdom, the mandate of the committee is binding (15).
- **Defining decision criteria**: While many countries assess similar types of evidence (burden of disease, availability of treatments, prevalence, efficacy, safety, clinical novelty, public health benefit, cost effectiveness, and ethical considerations), the specific criteria used, their level of provision and requirement, and the way they are incorporated (e.g., explicitly versus implicitly) varies across countries, with their relative weighting remaining generally unknown, as reported in a systematic review of HTA in eight European countries (14). Almost all countries look at clinical benefits or effectiveness. In Sweden, an “ethical platform” is translated into principles that guide national and local health decisions. However, highly autonomous city councils sometimes use those principles differently (15). While most countries have a well-defined set of criteria, criteria for decision making are not always explicitly defined, for example, in Slovenia, Japan, and Luxemburg (20). Some countries - but not all - include an assessment of cost effectiveness in their criteria including Brazil, Thailand, Canada, United Kingdom, Scotland, and Australia. Some criticism has been made of an assessment based purely on cost per quality-adjusted life year (QALY), and critics have highlighted the importance of “capturing social value” (14). Israel, like France and Germany, does not base the assessment on cost effectiveness. The Israeli process is not motivated by cost alone, but its strength is the integration of all important elements, including clinical benefit, cost, social, and ethical issues, to conform to the strictest ethical and legal considerations.
- **Selecting health technologies for HTA**: Israel has an “open procedure” by inviting applications for new technologies, similar to Thailand, Brazil, and the United Kingdom; other countries have a “closed” procedure which is not made public or a “targeted” procedure within defined fields, such as Ukraine.
  Some countries do not use HTA as a formal part of decision making for all types of medical technologies, pharmaceuticals, and devices, while some demonstrate partial use. While other countries also have a single institution, as in Israel, which carries out an assessment of all new medical technologies (e.g., Netherlands and Spain), countries, like France and Greece, have a separate assessment body for each type of technology (pharmaceuticals, procedures, and medical devices) (20).
  One of the distinctive features of the Israeli process is the comparison of drugs and technologies being considered, while some other countries examine technologies one at a time. The Israeli basket committee examines all the new technologies simultaneously over a designated period each year, rating their relative merits. Other countries with a similar process are the Australian Pharmaceutical Benefits Plan and New Zealand’s Pharmac.
- **Communication and appeal:** Communication is important for the implementation of decisions. About half of OECD countries publish information on assessments and recommendations for coverage for all or some technologies. In the Czech Republic, Denmark, France, and Sweden, such information is published on a dedicated website and easily accessible, as in Israel (20). Fewer countries publish the rationale for decisions. The US Institute for Clinical and Economic Review (ICER) makes its meetings public and live streams and archives them on the ICER website to ensure the legitimacy and uptake of its recommendations (21). The UK’s NICE also opens some committee meetings to the public (e.g., during evidence presentation), while sessions on the deliberation of recommendations remain closed. Israel publishes meeting transcripts and protocols.
  NICE summarizes and publishes key evidence, its argumentation, and its preliminary recommendation. Comments are considered in a second committee meeting, after which a “final appraisal determination” is issued, to which appeals can be made within 15 working days (15).
  Stake holders are informed of the recommendations in France (sent to the government and sponsors and published on the website), Thailand (recommendations adjusted to the target audience and published in diverse media platforms), Canada (a single digital newsletter issued weekly), United Kingdom (sent to NHS, patient, and the public), and Australia. In Brazil and Germany, recommendations are only published online (15).
  In Israel, the detailed list of recommendations which is the product of the Israeli decision-making process is published in its entirety in official MOH publications, including the name of the active ingredient for medications and a list of indications, the target population, and so forth. This explicit list facilitates implementation and avoids dilemmas of interpretation. Furthermore, technologies not approved can be resubmitted at the next cycle.
- **Monitoring and evaluation:** Collecting and analyzing data in order to judge if expectations were met and if improvement measures are needed. Scotland continuously evaluates how the Scottish Medical Consortium involves patients and the public and which are the best practices for public involvement (15). In Israel, monitoring is conducted both internally (at the MTIR) and by the government and national health council. Each year, there are consultations with pharmaceutical companies, patient organizations, and representatives from the Ministry of Health for continuous evaluation of the process. A budgetary evaluation is also conducted ensuring that the budget for the recommendations for updating the list of services is met and not exceeded.

## Discussion

Israel, like all countries, faces increasing tension between the abundance of technological advances that could improve patients’ health and the budgetary constraints that require a selection process, deciding which new drugs or medical devices will be publicly funded (1).

Israel’s GDP per capita in 2021 (44052 USD) was below the OECD average (49036 USD) (22). Expenditure on health - 2903 USD per capita in 2019 - was relatively low in Israel, compared with the OECD average 4087 USD (2;23). Health spending as a proportion of gross domestic product is also lower in Israel (7.8 percent) compared with the OECD average (9.5 percent) (latest figures from 2021) (24). Despite these figures, the Israeli healthcare system achieves good health outcomes for its citizens: international comparison demonstrates life expectancy at birth among the highest at a mean of 82.6, compared with the OECD average of 81.0 in 2021 (25). Potential years of life lost and cancer mortality rates were the lowest in 2020 (26). In 2021, infant mortality rates were 2.5 per 1000 live births in Israel, compared with 3.1, 3.6, and 5.4 per 1000 live births in Germany, the United Kingdom, and the United States, respectively (27). In 2019, the proportion of adults rating their own health as good or very good was 73.6 percent in Israel and 68.5 percent OECD average (28). Israel is also rated as a “leader” based on the technology achievement index which evaluates the technological performance and achievements of countries (29).

The annual update of the national list of reimbursed health services is unique and regarded as one of the contributors to Israel’s achievement of relatively good health for less money, described in the Lancet as “*a ground-breaking approach”* (8). Decision makers can set the yearly budget according to the economic situation, and the process can adapt to budgetary changes.

The rapid and efficient flow of the prioritization process in Israel results from several characteristics, including the early involvement of stake holders, which increases their involvement and commitment to the process. This national process is built on cooperation between governmental and public bodies. The two-phase process, in which the first body assesses the new technology, and a separate multi-sector committee is responsible for the prioritization and final decision making, ensures a fair prioritization. Furthermore, all proposed technologies are evaluated on an equal footing, with the applicant’s identity kept confidential from the committee to avoid prejudice. This process is designed to enable quick assessment and inclusion of the selected technologies in the publicly funded list so that patients can access treatment as quickly as possible. Participation of all four HMOs in the decision-making process facilitates rapid inclusion of new technologies in the services provided to their members, within approximately 2 weeks of the committee’s decision. The money follows the decision - funds are distributed according to the committee’s recommendations, to enable immediate implementation. The involvement of relevant stake holders in the process facilitates speedy implementation. Within 1–2 weeks of the final decision, the government approves the list of reimbursed services, and the MOH director general instructs the HMOs to provide the approved technologies to patients. Uptake of the newly approved technologies by the HMOs is monitored, allowing modification to decisions or reutilization of the budget to other technologies as appropriate.

Over the years, a dedicated budget for new technologies, risk-sharing agreements, and a proportion included without an additional allocated budget due to offsetting of outdated technologies or other savings have enabled the inclusion and early adoption of some “blockbusters”, that is, high-cost technologies. Among other examples, CAR-T immunotherapy was included as a pioneering treatment for leukemia and lymphoma and Spinraza™ for spinal muscular atrophy patients. Israel was also the first country to adopt pembrolizumab (Keytruda) as a front line therapy for unresectable or metastatic melanoma.

The transparency of the entire process (including committee meetings open to the press, most information concerning applications, discussions and decisions published online, and appealing process) contributes to its legitimacy and acceptance by the judicial system. A 2013 report by the OECD described the Israeli approach as: “admirably formal and transparent” (30).

Over the years, the process was supported by the Israeli judicial system, which defended its fairness and lack of prejudice or outside influence. Furthermore, the involvement and investment of all relevant stake holders in the process increase its public acceptability.

**The Israeli model faces several challenges:**

1. Maintaining the quality of the list of reimbursed health services, that is, the challenge of including and funding new and cutting-edge technologies in the face of rising costs of technologies and a rising number of applications, within a limited budget which does not increase proportionally.
2. The involvement of public representatives and healthcare professionals in the process may create a conflict of interests, for example, patient advocates who try to lobby for a technology that could benefit themselves or a group they advocate for. Physicians, who are public committee members, have to withdraw their vote when a certain technology which presents a conflict of interest to them is under consideration. To reduce such conflicts, all participants in the public committee receive yearly guidance from the legal department of the MOH on possible conflicts of interest. All participants are required to fully disclose any conflict and are instructed as to the limitations of their role in the committee and as to what they are allowed to discuss with stake holders outside the committee. Furthermore, to aid transparency, conflicts of interest of committee members are declared online on the MOH website.
3. A predetermined timetable reduces the flexibility required to deal with “urgent” situations (breakthrough technologies) that emerge during the year since such technologies cannot enter the process in its midst. In such cases, the compassionate use of treatments or access through clinical trials may be made available until committee authorization is obtained.
4. Fluctuations in the budget allocated by the government for the process make long-term planning challenging.
5. An accumulating gap, created by the growth of technological advancement while the proportion of the public financing of healthcare expenditure has gradually declined, might be one of the factors contributing to the relative and absolute increase in out-of-pocket spending (31). The proportion of private financing has reached 38 percent, compared with an average of 26 percent in OECD countries (31). Between 1997 and 2014, private expenditure on healthcare increased from 3.9 to 5.9 percent of net household income (32).

## Conclusion

Despite a relatively small health budget, Israel has designed and implemented a process, now in place for 25 years, which allows for the public funding of a wide range of health technologies on a national level, providing cutting-edge treatment options for patients and maximizing the available budget. Despite an increasing number of applications for new technologies, the prioritization process successfully reaches a consensus each year on which technologies to fund within budget constraints. Over more than two decades, the guiding principles of the Israeli model have been maintained, while the process has evolved. Its evolution included the understanding of which data should guide the decision making; the consultation with professional experts and incorporating their input into the process; and increasing transparency and public involvement, which contributed to the high acceptability of the process. This accumulated experience led to the improvement of economic tools such as risk-sharing agreements, which allowed for a more efficient utilization of the budget, and databases that detect underutilization of adopted technologies allow a further increase in the number of approved technologies within budgetary constraints. The Israeli process is not motivated by cost alone, it derives its strength from the integration of all important elements including clinical benefit, cost, social, and ethical aspects. This transparent, fair process enables just allocation of resources while maximizing the adoption of novel technologies that contribute to an outstanding national level of health.
